# UAV-Based Image and LiDAR Fusion for Pavement Crack Segmentation

**DOI:** 10.3390/s23239315

**Published:** 2023-11-21

**Authors:** Ahmed Elamin, Ahmed El-Rabbany

**Affiliations:** 1 Department of Civil Engineering, Toronto Metropolitan University, Toronto, ON M5B 2K3, Canada; rabbany@torontomu.ca; 2 Department of Civil Engineering, Faculty of Engineering, Zagazig University, Zagazig 44519, Egypt

**Keywords:** crack detection, UAV, LiDAR, pixel-level, deep convolutional neural network

## Abstract

Pavement surface maintenance is pivotal for road safety. There exist a number of manual, time-consuming methods to examine pavement conditions and spot distresses. More recently, alternative pavement monitoring methods have been developed, which take advantage of unmanned aerial systems (UASs). However, existing UAS-based approaches make use of either image or LiDAR data, which do not allow for exploring the complementary characteristics of the two systems. This study explores the feasibility of fusing UAS-based imaging and low-cost LiDAR data to enhance pavement crack segmentation using a deep convolutional neural network (DCNN) model. Three datasets are collected using two different UASs at varying flight heights, and two types of pavement distress are investigated, namely cracks and sealed cracks. Four different imaging/LiDAR fusing combinations are created, namely RGB, RGB + intensity, RGB + elevation, and RGB + intensity + elevation. A modified U-net with residual blocks inspired by ResNet was adopted for enhanced pavement crack segmentation. Comparative analyses were conducted against state-of-the-art networks, namely U-net and FPHBN networks, demonstrating the superiority of the developed DCNN in terms of accuracy and generalizability. Using the RGB case of the first dataset, the obtained precision, recall, and F-measure are 77.48%, 87.66%, and 82.26%, respectively. The fusion of the geometric information from the elevation layer with RGB images led to a 2% increase in recall. Fusing the intensity layer with the RGB images yielded a reduction of approximately 2%, 8%, and 5% in the precision, recall, and F-measure. This is attributed to the low spatial resolution and high point cloud noise of the used LiDAR sensor. The second dataset crack samples obtained largely similar results to those of the first dataset. In the third dataset, capturing higher-resolution LiDAR data at a lower altitude led to improved recall, indicating finer crack detail detection. This fusion, however, led to a decrease in precision due to point cloud noise, which caused misclassifications. In contrast, for the sealed crack, the addition of LiDAR data improved the sealed crack segmentation by about 4% and 7% in the second and third datasets, respectively, compared to the RGB cases.

## 1. Introduction

Pavement cracks are the most prevalent type of road distress, which affects its lifetime. A crucial component of the maintenance mission is frequent and accurate crack detection. Sealed cracks represent another crucial aspect of pavement distress that demands thorough evaluation within the pavement management framework. Sealing cracks involves filling existing cracks in the surface layer of asphalt concrete pavement with sealant. Damage to these sealed cracks can adversely impact the visual appeal of the pavement, vehicle operation, and overall driving comfort [1]. Detecting pavement cracks and sealed cracks is crucial for road safety, preventing further damage, and prolonging pavement lifespan [2,3,4]. The traditional manual inspection of roads is not only time-consuming and labour-intensive but also subjective [5]. Different platforms have been adopted to achieve automated pavement inspection, such as survey vans, mobile mapping systems (MMS), and unmanned aerial vehicles (UAVs). These platforms are often equipped with cameras, laser scanners, ground penetrating radar (GPR), and light detection and ranging (LiDAR) [6,7,8].

Sealed cracks have received limited attention in the research domain. Few studies have specifically addressed detecting sealed cracks, and manual methods remain prevalent in engineering practices [1]. Sun et al. [9] enhanced the Faster R-CNN for identifying sealed crack bounding boxes. They employed a multi-model combination and multi-scale localization strategy to improve accuracy. Machine learning techniques have been employed to mitigate noise effects in crack detection by incorporating predefined feature extraction stages before model training. For example, Zhang et al. [3] developed a T-DCNN pre-classification to categorize cracks and sealed cracks in pavement images relying on tensor voting curve detection. However, their approach had limitations in detecting fine cracks, i.e., the detected cracks suffered from background noise and discontinuities. More recently, Shang et al. [1] proposed a sealed crack detection approach using multi-fusion U-net. The proposed approach outperformed seven state-of-the-art models, including U-net, SegNet, and DeepLabV3+. The achieved precision, recall, and F-measure were 79.64%, 91.59%, and 84.36%, respectively.

On the other hand, extensive research has delved into crack detection through various approaches and platforms. For example, Quintana et al. [10] used a single linear camera mounted on a truck connected to a tracking device consisting of an odometer and a global positioning system (GPS) receiver to geo-localize the captured images. Image processing was accomplished using a crack classification computer vision algorithm. The precision and recall values were between 80% and 90%. In the work of Kang and Choi [11], two 2D LiDAR units and a camera were used for pothole detection. The LiDAR data processing included noise reduction, clustering, line segment extraction, and gradient of data function. The video collected by the camera was processed through various stages, including brightness control, binarization, object extraction, noise filtering, and pothole detection. However, this approach processed the LiDAR and camera data separately and then combined the output to improve the results. Both [12,13] used the mobile laser scanning (MLS) system RIEGL VMX-450 for detecting asphalt pavement cracks, which is considered a high-cost system. In [12], their approach for crack detection included ground point filtering, high-pass convolution, matched filtering, and finally, noise removal. They demonstrated that their proposed method could detect moderate to severe cracks (13–25 mm) in an urban road. In [13], a semi-supervised 3D pavement crack detection algorithm was developed based on graph convolutional networks. The approach achieved 73.9% in recall and 71.9% in F-measure.

Recently, UAVs have been widely used in crack detection applications due to their versatility, low cost, and ability to be mounted with various sensors [14]. Several studies have used UAV-based images to detect cracks using deep learning [15,16,17,18,19,20,21]. For example, C. Feng et al. [15] used UAV images to detect cracks on a dam surface using a deep convolution network. Chan et al. [17] proposed a two-step deep learning method for the automated detection of façade cracks in building envelopes using UAV-captured images. Their approach, which involved a CNN model for classification and a U-Net model for segmentation, achieved high precision and recall. This, in turn, enhanced the reliability of detecting cracks and enabled comprehensive assessment of façade conditions for maintenance decisions. In [18], UAV-based images were used to detect cracks for bridge inspection. Moreover, [21] used UAV-based images for pothole recognition based on the You Only Look Once (YOLO) v4 classifier. They obtained roughly 90% crack classification accuracy. In [19], a support vector machine (SVM) was adopted to identify cracks from aerial UAV RGB images. The images were then classified into two categories, namely cracks and non-cracks. Their proposed approach achieved a 97% classification accuracy.

They also showed that shadows in the images could potentially be misclassified as a crack. Y. Pan et al. [22] compared three learning algorithms, namely SVM, artificial neural networks, and random forest (RF), to detect asphalt road pavement distress from multispectral images acquired by a UAV-based camera. They showed that RF has higher accuracy than the other two algorithms, with about 98% average accuracy.

Recently, computer vision and deep learning techniques have been effectively applied to automate crack classification and segmentation [4,23,24,25,26,27,28,29]. In particular, DCNN methods have shown better crack detection performance than traditional image processing methods [30]. Crack classification DCNNs output labels for the input images, which indicate the class of the whole image (i.e., cracked, non-cracked, fatigue cracks). In [4], a DCNN model that classified the input images into cracked and non-cracked was introduced. The model was composed of four convolutional layers, a maximum pooling layer, and finally, two fully connected layers. The precision and recall were approximately 86% and 92%, respectively. Li et al. [31] classified 3D images of pavement cracks using four DCNNs into five classes, namely non-crack, transverse crack, longitudinal crack, block crack, and alligator crack. The overall accuracies of the four were above 94%.

Whereas an image is classified as one unit in image classification techniques, each pixel in the image is labelled in semantic segmentation. Both [25,26] used crack segmentation techniques for post-disaster assessment purposes. Wang et al. [23] presented an innovative framework for automatic pixel-level tunnel crack detection using a combination of weakly supervised learning methods (WSL) and fully supervised learning methods (FSL). Their approach, which was validated on a large dataset, achieved an F-measure of 0.865. In [32], a supervised DCNN was proposed to learn the crack structure from raw images. Their network architecture contained four convolutional, two max-pooling, and three fully connected layers. All segmentation metrics (i.e., precision, recall, and F-measure) were around 90%. A feature pyramid and hierarchical boosting network (FPHBN) for crack segmentation was proposed in [33]. FPHBN was composed of four major components, namely bottom-up architecture, feature pyramid, side networks, and hierarchical boosting module. The bottom-up architecture was used for hierarchical feature extraction, while the feature pyramid was used for merging information to lower layers. The side networks were for deep supervision learning, and the hierarchical boosting module was to adjust sample weights. The authors created the CRACK500 dataset, which was divided into 1896 images for training data, 348 images for validation data, and 1124 images for test data. The network achieved an average intersection over union (AIU) of 48.9%. Both DeepCrack [34] and the fast pavement crack detection network (FPCNet) [35] utilized encoder–decoder architecture networks for crack segmentation. DeepCrack was trained on the collected dataset, which contained 260 images. The DeepCrack network reached an F-measure of over 87%. Meanwhile, FPCNet reached a 95% F-measure after being trained using the CFD dataset. The CFD dataset consisted of 118 images and was published in [36]. Jenkins et al. [37] proposed a DCNN based on U-net for crack segmentation. The U-net architecture was mainly composed of an encoder and a decoder [38]. The network was tested on the CFD dataset. The results were 92%, 82%, and 87% for precision, recall and F-measure, respectively. In [39], an enhanced U-net architecture was proposed, where the convolution blocks were replaced with residual blocks inspired by ResNet [40]. The proposed network was evaluated using the CFD data set and CRACK500 dataset. On the CFD dataset, the proposed network achieved 97%, 94%, and 95% for precision, recall, and F-measure. While on challenging datasets such as CRACK500, it achieved 74%, 72%, and 73% for precision, recall, and F-measure. In both datasets, this approach marginally outperformed other methods.

The use of a vehicle equipped with a camera or a LiDAR sensor would potentially affect the traffic flow due to the low speed needed to capture high-quality images or dense point clouds. In addition, mapping a large area or a multi-lane road would be complex and inefficient. None of the UAV-based studies addressed the use of low-cost LiDAR data for crack segmentation. LiDAR can directly acquire geometry information of the pavement and is not affected by illumination conditions. However, the image-based methods might potentially obtain a higher accuracy when compared to it [13].

In this paper, a UAS-based RGB images and LiDAR data fusion model is developed using an enhanced version of the U-net for pavement crack segmentation. The network architecture is developed and pretrained, and its hyperparameters are tuned. Transfer learning is used to achieve more accurate segmentation results. Both mechanical and solid-state LiDAR sensors are considered in this study. Moreover, two different pavement distresses are investigated, namely cracks and sealed cracks. The remainder of this paper is organized as follows: Section 2 introduces the proposed methodology, while Section 3 discusses the adopted system, data collection, and data processing. Section 4 presents the results, and finally, Section 5 draws some conclusions about this research.

## 2. Proposed Crack Segmentation Method

A georeferenced orthomosaic image was created for the study area using the acquired images. The LiDAR data were then processed, and the resultant georeferenced point cloud was utilized to produce a digital elevation model (DEM) representing the surface topography and intensity rasters, capturing the reflectance information. Subsequently, four different imaging/LiDAR combinations were generated, namely RGB, RGB + intensity, RGB + elevation, and RGB + intensity + elevation. In the absence of GNSS data, it becomes challenging to register the images and LiDAR data. In such scenarios, manual or automatic registration methods, as demonstrated in [41], can be employed to establish the necessary correspondence between the images and LiDAR data. Inspired by Residual Network (ResNet) [39], an enhanced U-net architecture was adopted in this research, whereby residual blocks were considered as opposed to convolution blocks. The ResNet architecture was developed to mitigate the vanishing gradient problem by utilizing residual connections.

**Figure 1:**
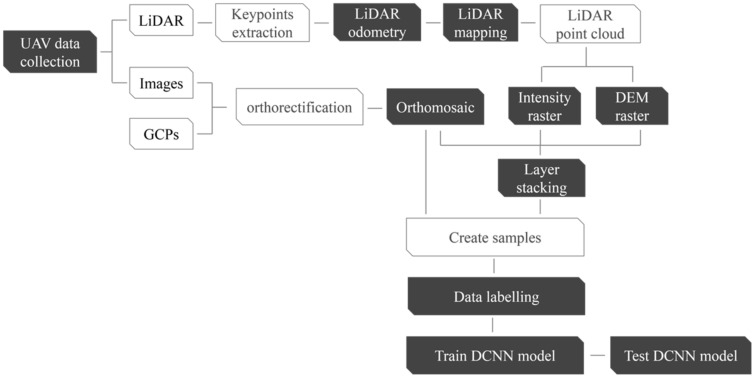
Proposed methodology flow chart.

A number of subset samples were generated from the images, composing the aforementioned four raster combinations for the cracked and sealed cracks dataset. Subsequently, all images were utilized for the DCNN training and testing, after which the network was pretrained using the CRACK500 dataset [30] in order to optimize the network weights and, thereby, performance. Transfer learning was then used to tune the network weights using all four combinations of both crack datasets. The methodology flowchart is shown in Figure 1.

### 2.1. Orthomosaic Image Creation

An orthomosaic image is composed of overlapped images to generate an undistorted 2D map that is corrected for scale and perspective [42]. Pix4D mapper software is a Photogrammetry software that generates an orthomosaic image from multiple images using photogrammetry principles [43]. First, key points are extracted and matched in the overlapped images utilizing the scale-invariant feature transform (SIFT) algorithm [44]. A sufficient overlap between images is needed to extract key points in multiple images. Pix4D software recommends a minimum of 75% overlap between images acquired from the nadir perspective to produce accurate orthomosaic images [42]. The triangulation technique is used to estimate camera poses and calibration parameters based on key points and ground control points (GCPs), after which a bundle adjustment is performed [45]. Then, key points are used to create DEM by triangulating them to 3D coordinates. Finally, the georeferenced orthomosaic image is created by projecting the DEM to image pixels.

### 2.2. Optimized LOAM SLAM

**Figure 2:**
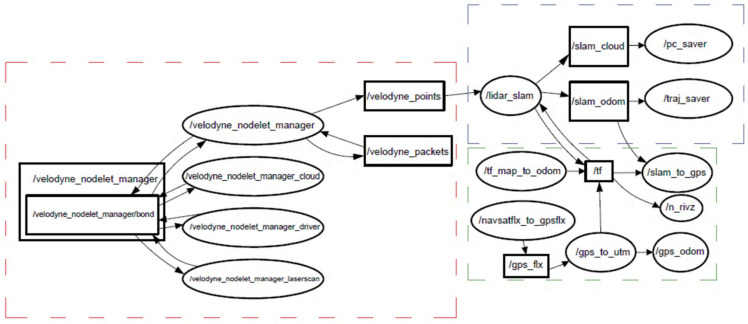
ROS computation graph of the Kitware LOAM.

The LiDAR point cloud of the first dataset was processed using an optimized simultaneous localization and mapping (SLAM) algorithm. The optimized SLAM was developed by Kitware on the basis of the LiDAR odometry and mapping (LOAM) algorithm [46,47]. The ROS computation graph of the Kitware LOAM is shown in Figure 2.

The optimized LOAM SLAM consists of three main subsequent steps. The first step is key point extraction, where the key points are categorized as planar and edge points based on curvature. The local smoothness of each point is defined based on its neighbouring points by

(1) $$c=\frac{1}{\left|S|.||X_{\left(k,i\right)}^{L}|\right|}||\sum_{j\in S,j\neq i}\left(X_{\left(k,i\right)}^{L}-X_{\left(k,j\right)}^{L}\right)||$$

where $X_{\left(k,i\right)}^{L}$ are the coordinates of point *i* belonging to the point cloud perceived during sweep *k* in the LiDAR coordinate system *L*, and *S* is its neighbouring points. Points with maximum curvature are considered edge points, while points with minimum curvature are considered planar points. In the second step, the iterative closest point (ICP) matching technique [48] is used in the LiDAR odometry phase to retrieve the LiDAR motion $T_{k+1}^{L}$ between successive frames.

(2) $$T_{k+1}^{L}={\left[\begin{array}{c} t_{x},t_{y},t_{z},\theta_{x},\theta_{y},\theta_{z} \end{array}\right]}^{T}$$

where *t_x_*, *t_y_*, *t_z_* are translations, and *θ_x_*, *θ_y_*, *θ_z_* are rotation angles with respect to the previous frame. Depending on feature type, point-to-line distance *dε* or point-to-plane distance *dH* can be computed as

(3) $$d_{\varepsilon }=\frac{\left|({\tilde{X}}_{\left(k+1,i\right)}^{L}-{\bar{X}}_{\left(k,j\right)}^{L})\times ({\tilde{X}}_{\left(k+1,i\right)}^{L}-{\bar{X}}_{\left(k,l\right)}^{L})\right|}{\left|{\bar{X}}_{\left(k,j\right)}^{L}-{\bar{X}}_{\left(k,l\right)}^{L}\right|}$$

(4) $$d_{H}=\frac{\left|\begin{array}{c} \left({\tilde{X}}_{\left(k+1,i\right)}^{L}-{\bar{X}}_{\left(k,j\right)}^{L}\right)\\ \left({\bar{X}}_{\left(k,j\right)}^{L}-{\bar{X}}_{\left(k,l\right)}^{L})\times ({\bar{X}}_{\left(k,j\right)}^{L}-{\bar{X}}_{\left(k,m\right)}^{L}\right) \end{array}\right|}{\left|({\bar{X}}_{\left(k,j\right)}^{L}-{\bar{X}}_{\left(k,l\right)}^{L})\times ({\bar{X}}_{\left(k,j\right)}^{L}-{\bar{X}}_{\left(k,m\right)}^{L})\right|}$$

where ${\tilde{X}}_{\left(k+1,i\right)}^{L}$, ${\bar{X}}_{\left(k,j\right)}^{L}$, ${\bar{X}}_{\left(k,l\right)}^{L}$, ${\bar{X}}_{\left(k,m\right)}^{L}$ are the coordinates of points *i*, *j*, *l*, m in *L*, respectively. The geometric relationship between an edge point and the corresponding edge line can be described as follows:

(5) $$f\varepsilon (X_{\left(k+1,i\right)}^{L},T_{k+1}^{L})=d\varepsilon$$

The geometric relationship between point-to-plane distance is described as follows:

(6) $$fH(X_{\left(k+1,i\right)}^{L},T_{k+1}^{L})=dH$$

By stacking both equations for each feature point, the following nonlinear function is obtained:

(7) $$f(T_{k+1}^{L})=d$$

Finally, in the third step, the current frame is projected and matched with the existing map to refine the recovered motion.

### 2.3. DCNN Architecture, Hyperparameters, and Performance Metrics

**Figure 3:**
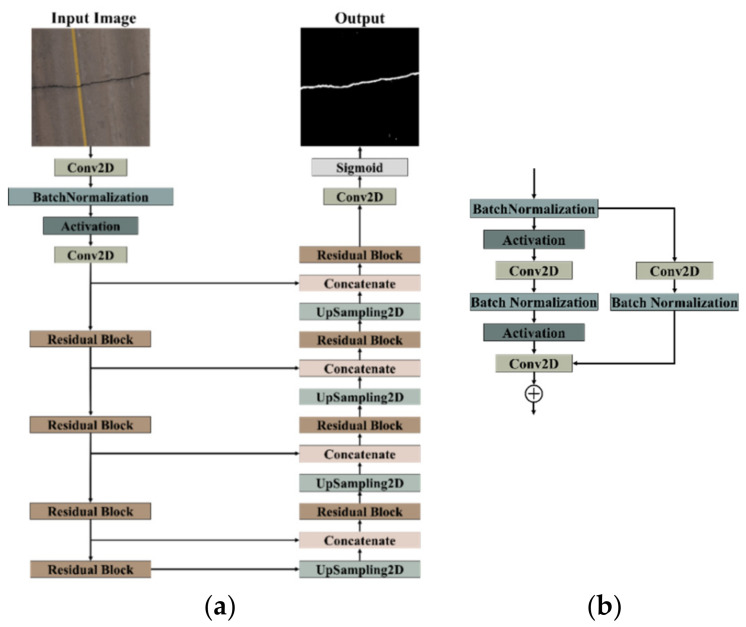
(**a**) The network architecture. (**b**) The residual block.

The architecture of the employed DCNN is a refined iteration of the U-net [38], as introduced by [39], chosen for its demonstrated excellence in crack semantic segmentation [39]. The adaptation involves substituting convolution blocks with residual blocks, maintaining the U-net structure with an encoder for feature extraction and a decoder for upsampling operations, as illustrated in Figure 3. The utilization of a 2D convolutional layer with a 7 × 7 kernel size and a stride of 2 serves the purpose of capturing broader contextual information within the input data, contributing to enhanced feature extraction and reduced noise. The incorporation of the dice coefficient loss function addresses the challenge of class imbalance between cracks and the background [49], providing a nuanced optimization approach. Additionally, the selection of the AMSgrad optimizer [50] is driven by its capacity to overcome certain limitations associated with the conventional Adam optimizer, ensuring stable and effective optimization during the training process. These considerations are strategically made to elevate the model’s proficiency in accurately segmenting pavement cracks across diverse scenarios.

(8) $$m_{t}=\beta_{1}m_{t}{}_{-1}+(1-\beta_{1})g_{t}$$

(9) $$v_{t}=\beta_{2}v_{t}{}_{-1}+(1-\beta_{2})g_{t}{}^{2}$$

(10) $$v_{t}=max({\overset{⌢}{v}}_{t}{}_{-1},v_{t})$$

(11) $$\theta_{t+1}=\theta_{t}-\frac{\eta }{\sqrt{{\overset{⌢}{v}}_{t}}+\epsilon }m_{t}$$

where $m_{t}$ is the first moment, $v_{t}$ is the second moment, $g_{t}$ is the gradient, $\theta_{t}$ is the parameters update, and η is the learning rate.

The hyperparameters $\beta_{1}$ and $\beta_{2}$ were set as the default values 0.9 and 0.999, respectively, whereas the epsilon number (ε) was set as 10^−7^, as usually recommended in practice [50]. These hyperparameters were carefully chosen based on empirical studies and established best practices in the field of deep learning optimization. A momentum value of 0.9 was employed to ensure stable convergence, preventing oscillations during training, while an exponential decay rate of 0.999 and an epsilon value of 10^−7^ were selected for efficient and precise updates to the model parameters, ensuring both numerical stability and accurate optimization. A learning rate schedular was utilized as a replacement for a constant learning rate. It changes the learning rate gradually upon the loss plateauing or after a specific number of epochs. As a result, the optimum convergence is achieved by the end of training. The training data size was increased with the aid of a random image augmentation technique. The augmentations included random rotations and flips using the Albumentations library [51].

Precision (Pr), recall (R), and F-measure metrics were considered to assess the segmentation performance. The precision is the ratio between the correctly classified pixels as cracks and all pixels predicted as cracks. The recall is the ratio between the correctly classified pixels as cracks and the total ground truth crack pixels. F-measure represents the weighted mean of precision and recall. This metric is used in the case of severe class imbalance. The performance metrics formulas are depicted below:

(12) $$\mathrm{Pr}=\frac{TP}{TP+FP}$$

(13) $$R=\frac{TP}{TP+FN}$$

(14) $$F-measure=\frac{2\times Pr\times R}{\mathrm{Pr}+R}$$

where TP represents the true positive, which indicates that the predicted crack pixel is also a crack pixel in the ground truth; FP represents the false positive, which indicates that the predicted crack pixel is a background pixel in the ground truth; FN is the false negative, which indicates that prediction result is a background pixel, but the ground truth is a crack pixel. The network was developed and executed using Tensorflow and Keras [52,53].

## 3. Data Collection and Preprocessing

**Figure 4:**
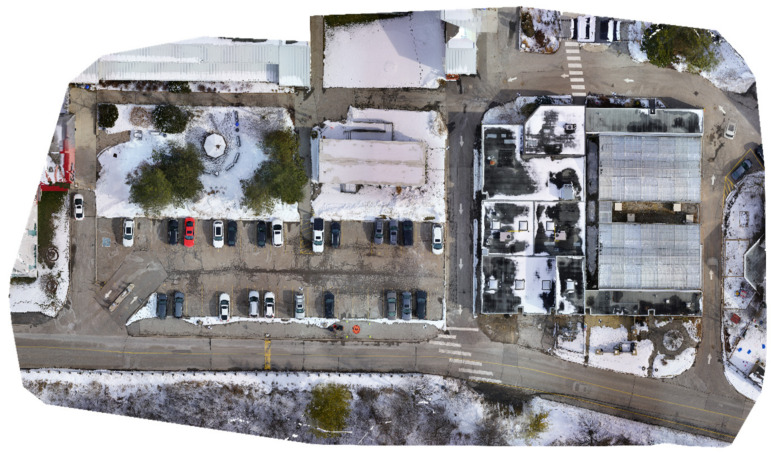
Orthomosaic image of the first dataset area [56].

Three data sets were collected in this study using two different UASs. In the first dataset, the UAS was a DJI Matrice 600 Pro with approximately 25 m flight height, with onboard sensors comprising a Sony α7R II RGB camera [54], a Velodyne Puck mechanical LiDAR sensor [55], and Applanix APX-15 GNSS/IMU board. For more details about the system and sensor configurations, the reader is referred to [56]. The total number of collected points was about 14 million points. The number of acquired RGB images was 243 images. Twelve GCPs were established in the study area. The targets’ coordinates were precisely determined to the centimeter-level accuracy using a GNSS receiver operating in real-time kinematic (RTK) mode. Using Pix4D mapper software, four GCPs were used to rectify the images in order to generate a georeferenced orthomosaic image, while the remaining GCPs were used as checkpoints. Figure 4 [56] presents the georeferenced orthomosaic image.

**Figure 5:**
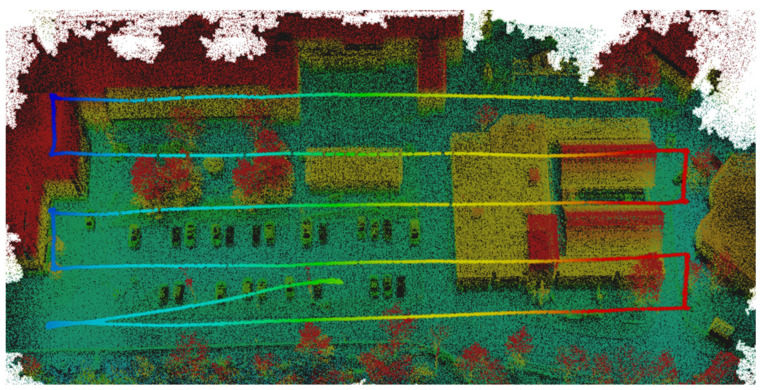
LiDAR SLAM trajectory of the first dataset.

**Figure 6:**
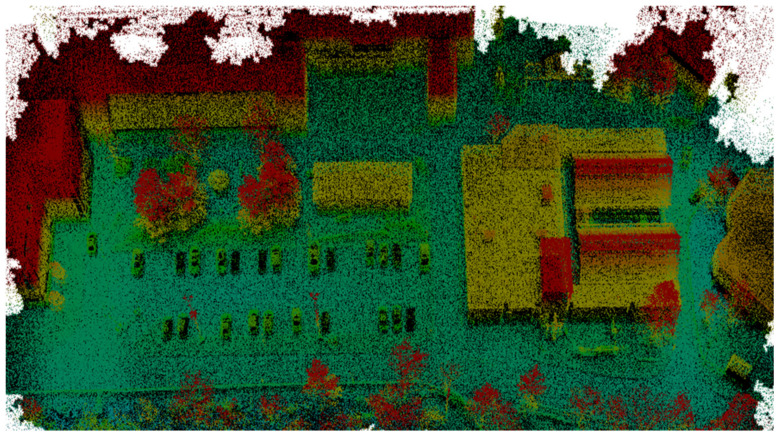
First dataset LiDAR point cloud generated using LOAM SLAM.

**Figure 7:**
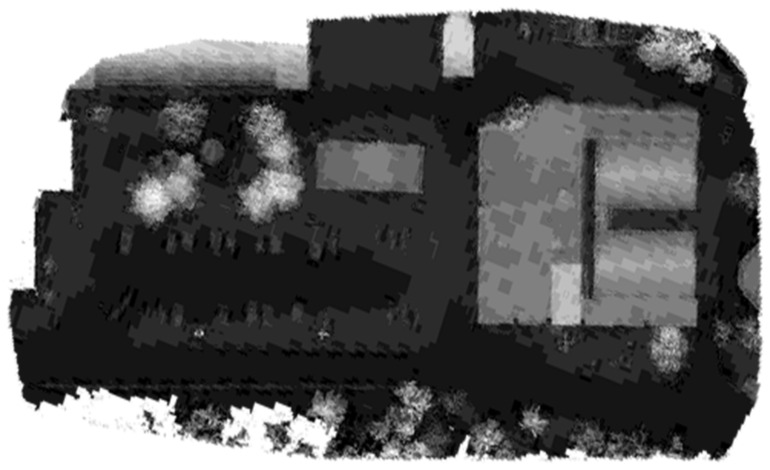
First dataset DEM raster created using the mechanical LiDAR point cloud [56].

**Figure 8:**
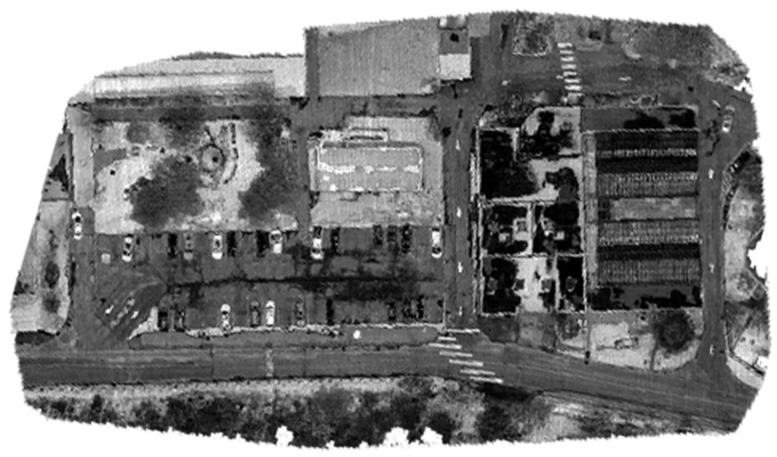
First dataset intensity raster created using the mechanical LiDAR point cloud [56].

The optimized LOAM SLAM algorithm was implemented to process the LiDAR data. The SLAM trajectory and the created point cloud are presented in Figure 5 and Figure 6, respectively. The LiDAR point cloud was georeferenced using the GCPs and then processed to create a digital elevation model (DEM) and intensity rasters using ArcGIS software, as shown in Figure 7 and Figure 8, respectively [56].

**Figure 9:**
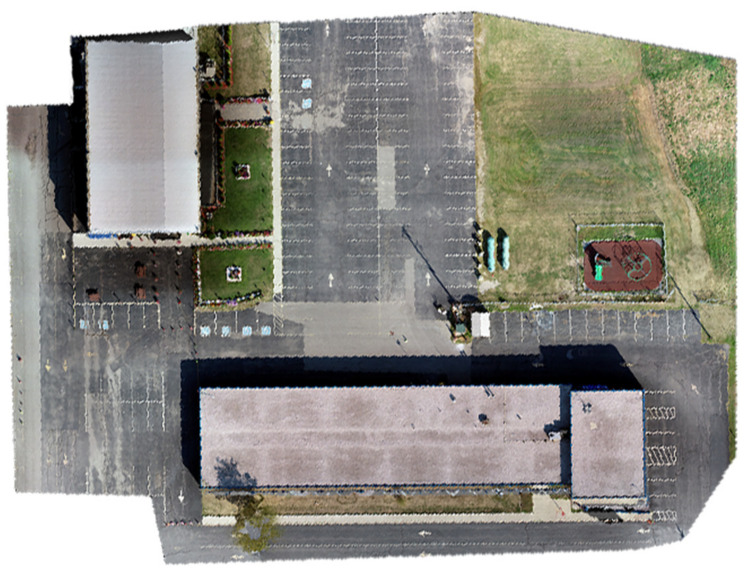
Orthomosaic image of the second dataset area.

**Figure 10:**
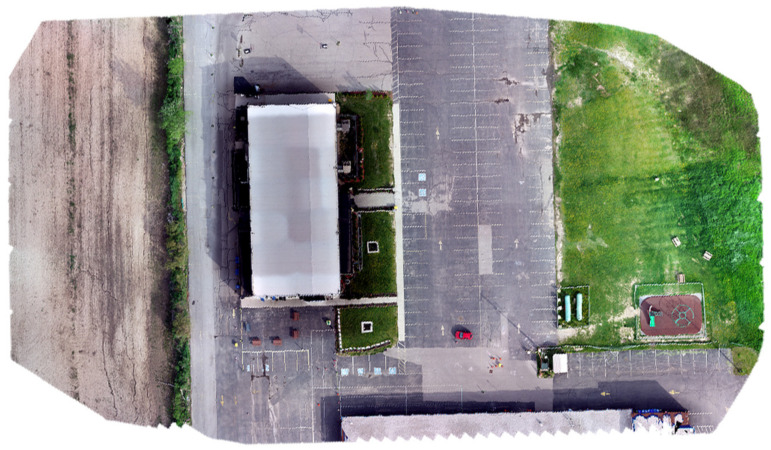
Orthomosaic image of the third dataset area.

**Figure 11:**
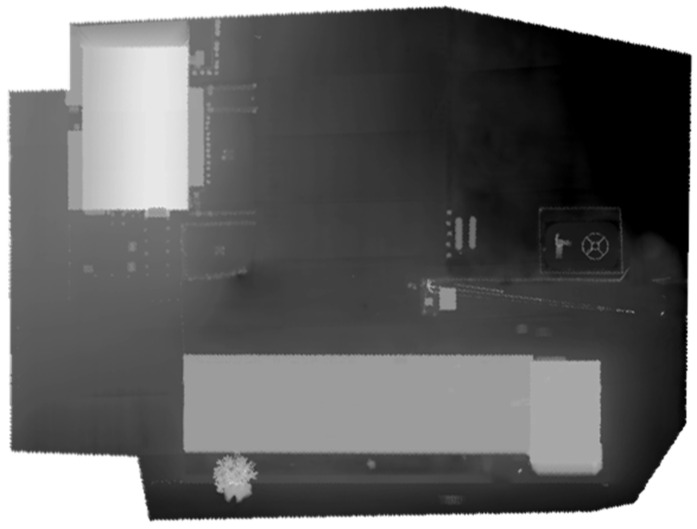
Second dataset DEM raster created using created using the solid-state LiDAR point cloud.

**Figure 12:**
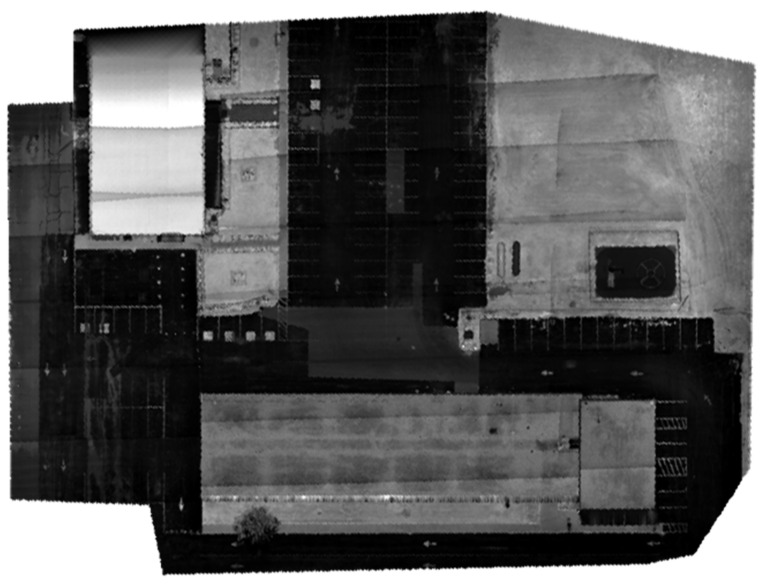
Second dataset intensity raster created using the solid-state LiDAR point cloud.

**Figure 13:**
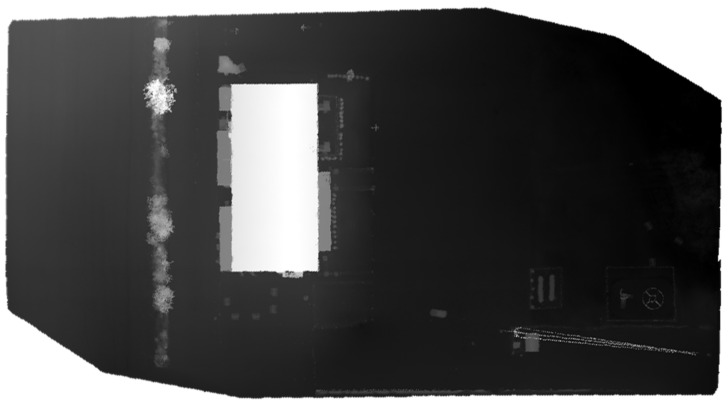
Third dataset DEM raster created using the solid-state LiDAR point cloud.

**Figure 14:**
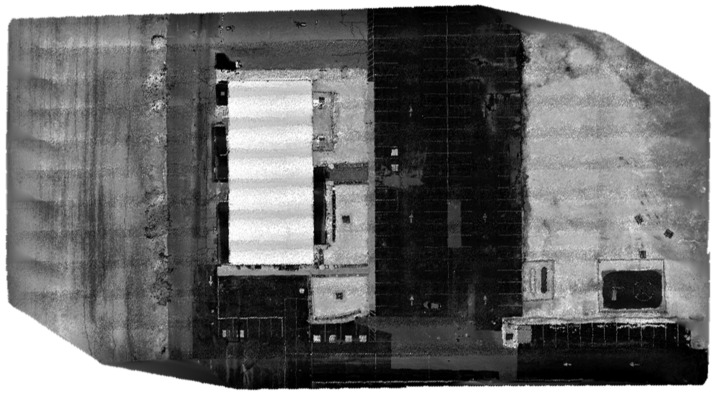
Third dataset intensity raster created using the solid-state LiDAR point cloud.

The second and third datasets were acquired using a DJI Matrice 300 RTK UAV equipped with a Zenmuse L1 system. The latter integrates a Livox solid-state LiDAR, an IMU, a GNSS receiver, and an RGB camera [57]. The second dataset was acquired at a flight altitude of 25 m, while the third dataset was obtained at an altitude of 19 m. This deliberate variation in flight height was undertaken in order to evaluate the impact of elevation on the accuracy of crack segmentation. For the second dataset, the total number of collected points using the solid-state LiDAR was about 123 million, and the number of acquired images was 224 using the RGB camera. In the third dataset case, approximately 340 million points were gathered through the solid-state LiDAR, accompanied by the acquisition of 548 images using the RGB camera. For both datasets, the precise drone path was determined to centimeter-level accuracy using GNSS RTK, which was used to georeference the collected data. Similar to the first case study, the geotagged images were processed to form a georeferenced orthomosaic image, as presented in Figure 9 and Figure 10 for the second and third datasets, respectively. The solid-state LiDAR data were processed using the DJI Terra software to generate a georeferenced point cloud of the study area. Then, similar to the first dataset, ArcGIS software was utilized to process the point cloud to generate DEM and intensity rasters, as shown in Figure 11, Figure 12, Figure 13 and Figure 14 for the second and third datasets, respectively.

Four imaging/LiDAR combinations were considered for the three datasets, namely RGB, RGB + intensity, RGB + elevation, and RGB + intensity + elevation. The first case (RGB) featured the use of the colored orthomosaic image. The second case (RGB + intensity) stacked the intensity and orthomosaic image using ERDAS IMAGINE software [58]. In the third case (RGB + elevation), the elevation raster was stacked to the RGB orthomosaic image as a fourth channel. In contrast, in the fourth case (RGB + intensity + elevation), the intensity, elevation, and the orthomosaic image were stacked.

To train the model, 24 crack samples of the first dataset were formulated as subset images of each of the four combinations. For the second and the third datasets, two types of pavement distress were detected, namely cracks and sealed cracks. Similar to the first dataset, subset images for each of these categories were created for each of the four combinations. A total of 20 sample images were created for each category of cracks and sealed cracks in the second dataset, and 30 images were generated for each category in the third dataset. The samples from each dataset were divided into training, validation, and testing sets, as illustrated in Table 1. The training sets were employed to train the DCNN, and the validation sets were used to fine-tune the model’s performance during training. It was ensured that the model had no prior exposure to the testing sets throughout the training process. The testing sets were employed exclusively to assess the model’s performance. The used testing sets exhibit a wide range of characteristics, including variations in crack shape, orientation, and background details, which differ significantly from the training and validation sets. This diversity serves to rigorously evaluate the model’s capacity to generalize across various features and real-world conditions.

**Figure 15:**
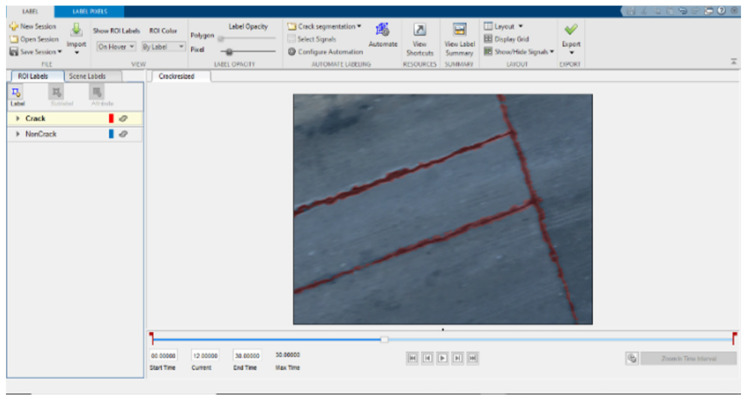
Labelling crack pixels using Matlab software.

**Figure 16:**
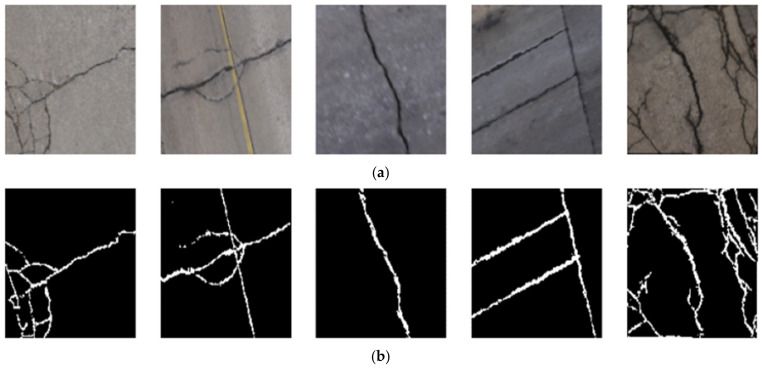
Crack samples of the first dataset: (**a**) pavement crack images and (**b**) ground truths.

**Figure 17:**
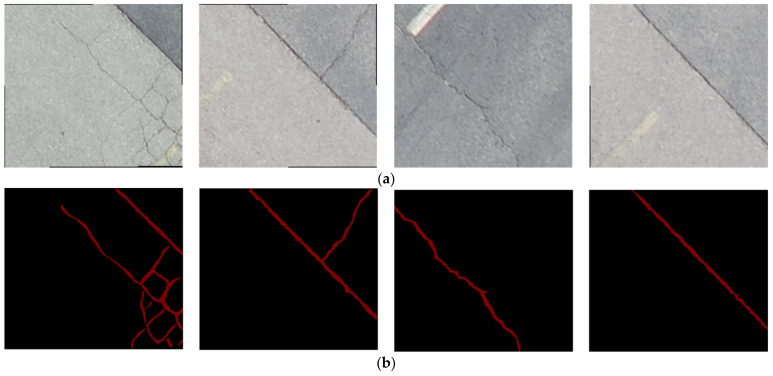
Crack samples of the second dataset: (**a**) pavement crack images and (**b**) ground truths.

**Figure 18:**
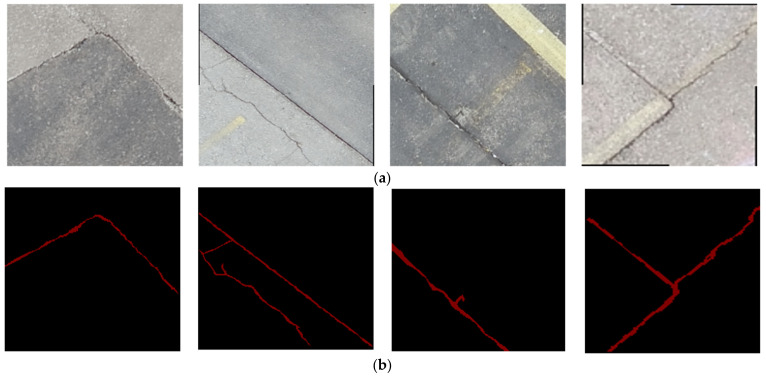
Crack samples of the third dataset: (**a**) pavement crack images and (**b**) ground truths.

**Figure 19:**
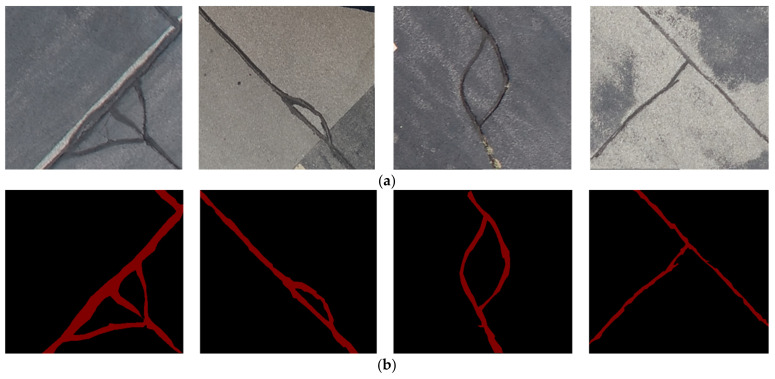
Sealed crack samples of the second dataset: (**a**) pavement crack images and (**b**) ground truths.

**Figure 20:**
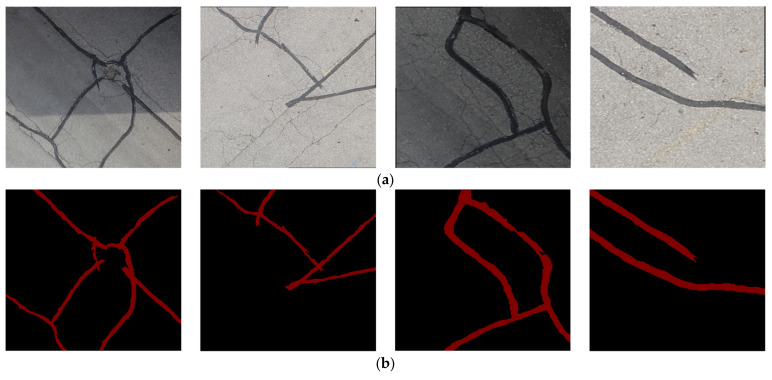
Sealed crack samples of the third dataset: (**a**) pavement crack images and (**b**) ground truths.

The targeted pavement distress grade in this research is moderate to severe distress (13–25 mm). To enhance the efficiency of the data labelling operation, a two-step automated ground truth labelling process was implemented using Matlab’s ground truth labeller [59]. In the first step, automatic ground truth labelling was performed using a pre-trained semantic segmentation algorithm, specifically DeeplabV3 [60], with ResNet as the base network, ensuring precise crack detection across images. The second step involved a manual verification process, allowing for minor manual adjustments. This manual review ensured the accuracy and reliability of the generated ground truth data. Figure 15 illustrates the process of labelling crack pixels using Matlab. The collected pavement distresses were challenging due to their different types and shapes and complex textures, including irregular patterns, shadows, or surface imperfections. Additionally, blurred backgrounds can pose challenges because the boundaries of cracks might not be distinct. Examples of pavement crack images of the first, second, and third datasets and their ground truth are presented in Figure 16, Figure 17 and Figure 18, respectively. Examples of the sealed crack images of the second and third datasets are shown in Figure 19 and Figure 20, respectively.

## 4. Results and Discussion

**Figure 21:**
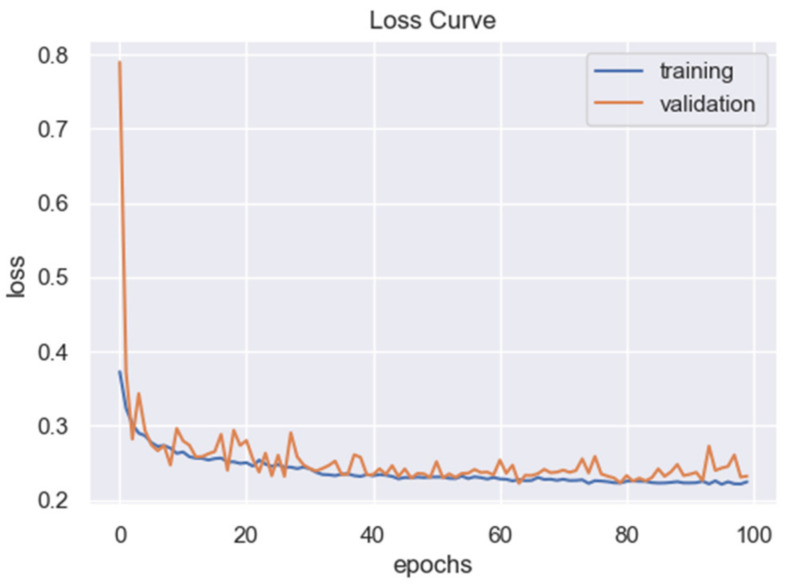
Training and validation loss curves for the pre-trained network.

**Figure 22:**
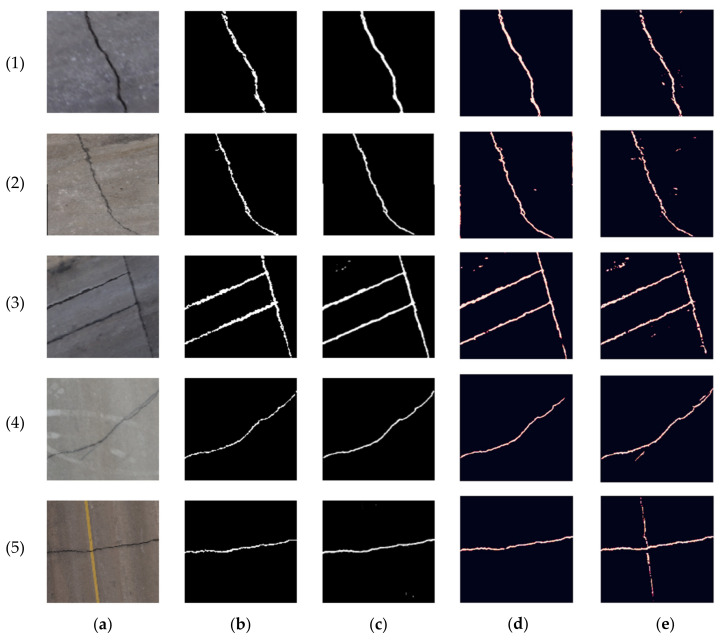
Prediction results comparison of our network with U-net and FPN using the RGB case of the first dataset crack samples: (**a**) pavement crack images, (**b**) ground truths, (**c**) our network, (**d**) U-net, (**e**) FPHBN.

The obtained segmentation accuracy was relatively low after training the network on the first dataset. This is mainly due to the limited size of the data. For example, the achieved precision, recall, and F-measure were 63%, 79%, and 70% in the RGB case, respectively. Therefore, to optimize the weights of the network and increase the segmentation accuracy, the CRACK500 dataset was used to pretrain the network. The training and validating loss curves are shown in Figure 21, and the precision, recall, and F-measure achieved by training the network on the CRACK500 dataset are presented in Table 2. After optimizing the network of the segmentation network, the pavement crack segmentation accuracy of the model was improved by about 10%. Before testing the network on the collected datasets, our model was compared with the U-net model [38] and the FPHBN model [33] using the RGB case of the first dataset. Our network outperformed both networks, as shown in Table 3. Figure 22 shows the predictions of the testing dataset for the three networks. It is noticeable that both U-net and FPHBN networks have some misclassifications of the pavement pixels as cracks. In addition, the FPHBN misclassified the shadow and the pavement marking as cracks in images 4 and 5, respectively.

**Figure 23:**
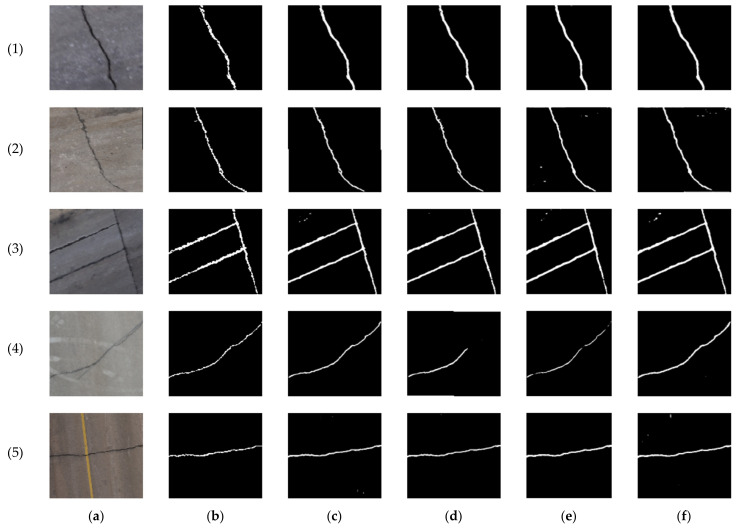
Prediction results comparison of the first dataset crack samples: (**a**) pavement crack images, (**b**) ground truths, (**c**) RGB, (**d**) RGB + intensity, (**e**) RGB + elevation, and (**f**) RGB + intensity + elevation.

Thereafter, the transfer learning technique was applied to train the network on the four combinations of the collected datasets. The RGB case serves as the baseline for all comparisons, allowing for an assessment of the enhancements in crack segmentation resulting from the incorporation of LiDAR data in comparison to image analysis alone. For the first dataset crack samples, as shown in Table 4, the achieved precision, recall, and F-measure in the RGB case were 77.48%, 87.66%, and 82.26%, respectively. In an attempt to improve the network performance, the LiDAR intensity layer was added to the RGB to formulate the RGB + intensity combination. The latter yielded a reduction of approximately 2%, 8%, and 5% in the precision, recall, and F-measure, respectively, in comparison with the RGB case. Instead, the elevation layer was added to the RGB to create the RGB + elevation combination. Through this combination, the obtained precision, recall, and F-measure were 72.41%, 89.00%, and 73.37%, respectively. Finally, both intensity and elevation layers were combined with the RGB to formulate RGB + intensity + elevation. Through this combination, the precision and F-measure were decreased by 6% and 4%, respectively, compared to the RGB case. The predictions of the testing dataset of the four different combinations for the crack samples of the first dataset are shown in Figure 23.

**Figure 24:**
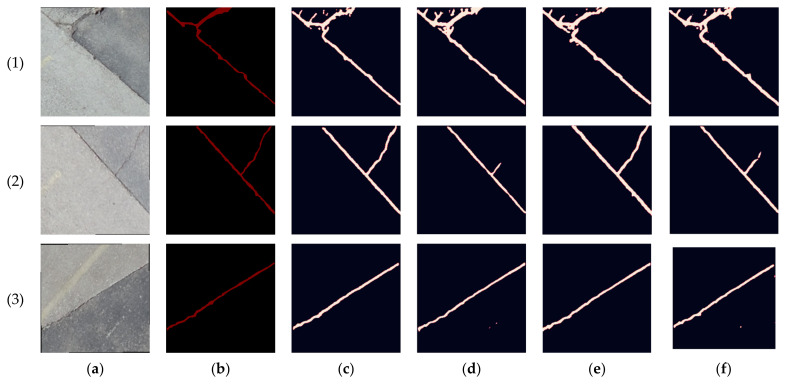
Prediction results comparison of the second dataset crack samples: (**a**) pavement crack images, (**b**) ground truths, (**c**) RGB, (**d**) RGB + intensity, (**e**) RGB + elevation, and (**f**) RGB + intensity + elevation.

The second dataset crack samples obtained similar results as shown in Table 5, where adding the intensity layer to the RGB decreased precision, recall, and F-measure by 11%, 2%, and 7%, respectively, in comparison with the RGB case. While adding the elevation increased the recall by about 2%, the RGB + intensity + elevation combination decreased the precision and F-measure by 10% and 5%. The increase in the recall value in the RGB + elevation combination for both datasets means that more crack details were detected by the network when geometric information represented in the elevation was added to the RGB images. In addition, some background pixels, such as stains, were classified as a crack in the third test image of the first dataset, showing the importance of adding geometric (depth) information to the images to avoid such confusion. The lower precision indicates that the network tends to misclassify background pixels as cracks. This is graphically noticeable, as shown in Figure 23 and Figure 24, where the predicted crack is thicker than its counterpart in the ground truth.

**Figure 25:**
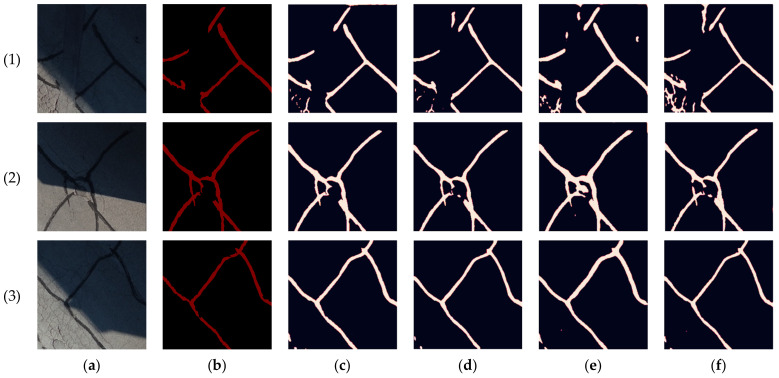
Prediction results comparison of the second dataset sealed crack samples: (**a**) pavement sealed crack images, (**b**) ground truths, (**c**) RGB, (**d**) RGB + intensity, (**e**) RGB + elevation, and (**f**) RGB + intensity + elevation.

For the sealed crack, considering the RGB case, the obtained precision, recall, and F-measure were 90.17%, 80.07%, and 84.82%, respectively, as shown in Table 6. Adding the intensity layer to the RGB and formulating the RGB + intensity combination improved the precision, recall, and F-measure by about 3%. As a substitute, the elevation layer was added to the RGB to create the RGB + elevation combination. Through this combination, the precision and F-measure were slightly improved compared to the RGB + intensity combination. Finally, by adding both the intensity and elevation layers to the RGB formulating the RGB + intensity + elevation combination, the precision was increased by 2%, and both the recall and F-measure were increased by 4% in comparison with the RGB case. Such an increase emphasizes the importance of image/LiDAR fusion, which helps mitigate the effects of shadows, as shown in the sealed crack images in Figure 25.

**Figure 26:**
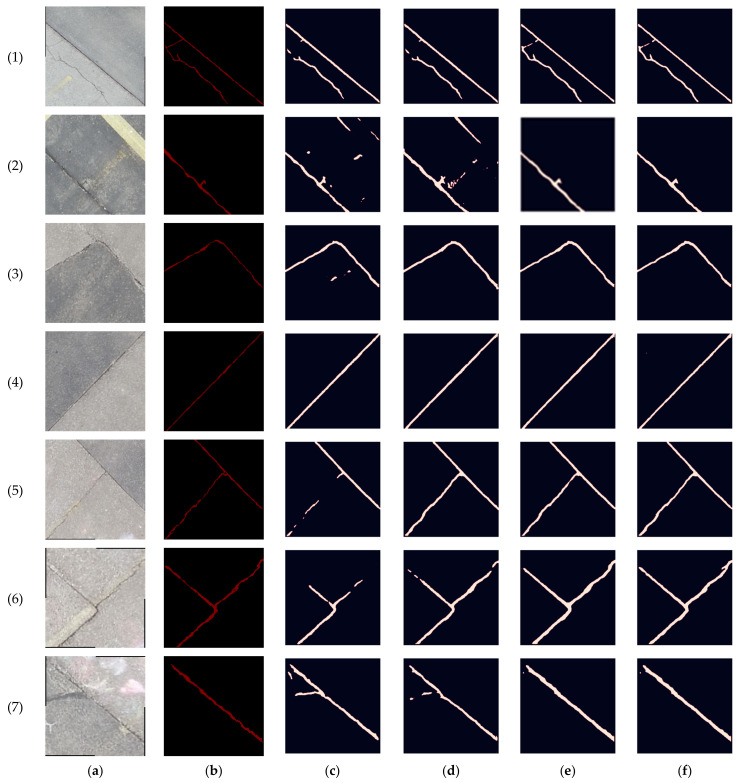
Prediction results comparison of the third dataset crack samples: (**a**) pavement crack images, (**b**) ground truths, (**c**) RGB, (**d**) RGB + intensity, (**e**) RGB + elevation, and (**f**) RGB + intensity + elevation.

In the third dataset, a lower flight height was investigated in order to capture higher-resolution data. Notably, as shown in Table 7, the segmentation performance of crack samples in the third dataset exhibited a slight improvement compared to the second dataset. In the RGB scenario, precision, recall, and F-measure increased by 1–2% in comparison with the RGB case of the second dataset. In contrast to the second dataset, incorporating the intensity layer with RGB in the third dataset facilitated the capture of finer crack details, leading to a notable 2% increase in recall in comparison with the RGB case. This enhancement is visually demonstrated in images 5 and 6 (Figure 26). Furthermore, adding the elevation to the RGB led to an even higher recall improvement of about 4% compared to the RGB case. However, in both RGB + intensity and RGB + elevation cases, there was a decrease in precision by 3% and 2%, respectively, compared to the RGB case. This reduction was attributed to either misclassification, as seen in image 2 (Figure 26), or the predicted crack being thicker than the actual crack in the ground truth. Nonetheless, it is noteworthy that the reduction in precision was considerably less than that of the second dataset, where the reduction reached 10%. This difference highlights the significantly enhanced LiDAR data resolution in this particular case.

**Figure 27:**
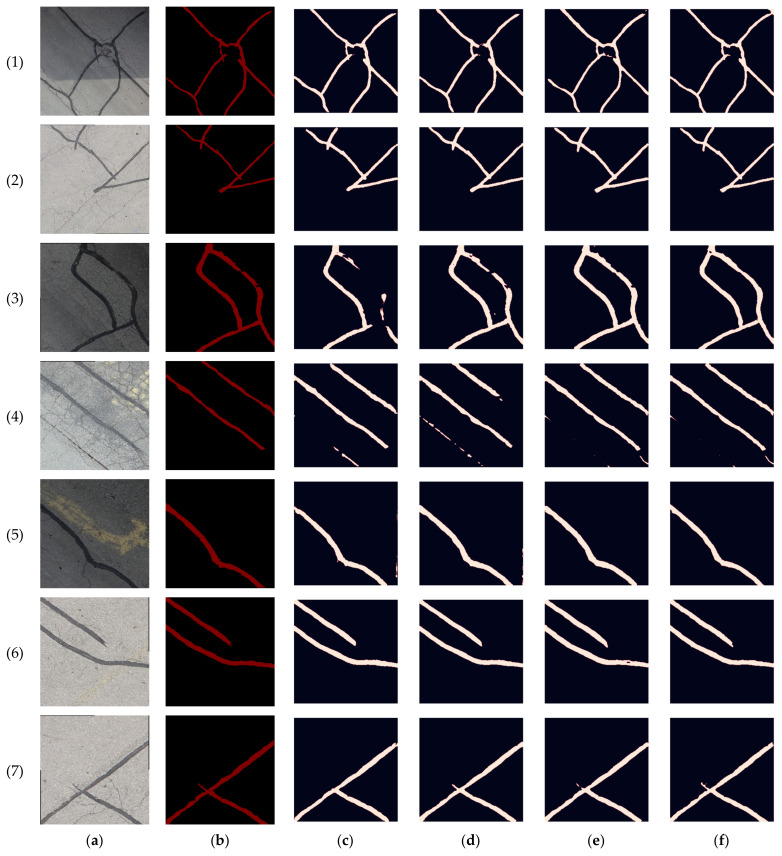
Prediction results comparison of the third dataset sealed crack samples: (**a**) pavement sealed crack images, (**b**) ground truths, (**c**) RGB, (**d**) RGB + intensity, (**e**) RGB + elevation, and (**f**) RGB + intensity + elevation.

In the context of sealed crack analysis, the results from the third dataset exhibited a similar trend to that of the second dataset, yet with a significant overall improvement in segmentation performance. Significantly, as shown in Table 8, the recall of the RGB case in the third dataset was improved by 5% compared to the RGB case of the second dataset. Integrating the intensity layer with the RGB, i.e., forming the RGB + intensity combination, resulted in consistent enhancements of approximately 1.5% in precision, 5% in recall, and 3% in F-measure. These results align with the outcomes of the second dataset. Remarkably, integrating the elevation layer with RGB, i.e., creating the RGB + elevation combination, led to a substantial 7% improvement in recall compared to the RGB case, surpassing the 3% improvement observed in the second dataset. This enhancement highlights the significant impact of the lower flight height, enabling the acquisition of higher-resolution LiDAR data, thus significantly improving semantic segmentation performance. Finally, the addition of both the intensity and elevation layers to the RGB increased the precision, recall, and F-measure by 2%, 7%, and 4%, respectively, compared to the RGB case. Such an increase emphasizes the importance of image/LiDAR fusion, enabling the capture of finer details of sealed cracks, as exemplified in image 3 (Figure 27), and the elimination of misclassifications, as demonstrated in image 4 of the same figure.

Integrating LiDAR data with the RGB images should enhance network performance significantly. This enhancement is primarily attributed to the intensity data, which help in differentiating materials and mitigating shadows, and the elevation data, which add crucial geometric information. However, the deterioration in segmentation accuracy metrics for crack samples in the first two datasets was essentially due to the limitations of the lower-grade LiDAR sensors. These sensors exhibit low spatial resolution and high point cloud noise, indicating that they might be better suited for detecting severe cracks or other forms of pavement distress, such as sealed cracks and potholes, rather than fine pavement cracks. Notably, in the third dataset, flying the UAV at a lower altitude enabled the capture of higher-resolution LiDAR data. Consequently, the incorporation of LiDAR data improved recall, indicating the detection of finer crack details. However, this improvement came at the cost of lower precision, primarily due to point cloud low resolution, leading to misclassifications.

In contrast, the proposed image/LiDAR data fusion significantly enhanced the segmentation of sealed cracks in the second dataset and demonstrated even greater improvements in the third dataset. This substantial enhancement emphasizes the critical importance of image/LiDAR fusion for pavement distress detection, particularly when aiming for accurate segmentation results in complex scenarios.

## 5. Conclusions

In this research, an advanced pavement distress segmentation model was developed, which fuses UAV-based high-spatial resolution camera images and low-cost LiDAR sensor data through a deep convolutional neural network. Two types of LiDAR sensors, a mechanical and a solid-state, were systematically evaluated across three datasets collected at varying flight altitudes. The first dataset contained pavement crack samples, while the second and third datasets included crack and sealed crack samples. Four imaging/LiDAR fused combinations were considered, which formed the basis for training and evaluating an enhanced U-net architecture.

In the evaluation stage, precision, recall, and F-measure were calculated for the test datasets. The fusion of LiDAR data resulted in decreased performance metrics for crack samples in the first two datasets due to limitations in lower-grade LiDAR sensors. These lower-grade LiDAR sensors are more suitable for detecting severe pavement distress than fine cracks. However, improved recall was observed in the third dataset, which was collected at a lower altitude, indicating enhanced crack detection when LiDAR data were fused with RGB images. This emphasizes the impact of lower flight height on the LiDAR data quality. In the context of sealed crack datasets within the second and third datasets, the integration of LiDAR showcased remarkable segmentation enhancement, emphasizing the pivotal role of image/LiDAR data fusion in pavement distress detection. This marks a significant advancement in precise and innovative pavement distress analysis.

## Figures and Tables

**Table 1:** Partitioning of samples into training, validation, and testing sets.

Dataset	Total Samples	Training	Validation	Testing
First	24	14	5	5
Second	30	12	5	3
Third	30	17	6	7

**Table 2:** The performance metrics for the pre-trained network on the crack500 dataset.

Dataset	Recall (%)	Precision (%)	F-Measure (%)
Crack500	77.54	81.71	79.57

**Table 3:** Comparison of the performance metrics of our network with U-net and FPN using the RGB case of the first dataset crack samples.

Dataset	Recall (%)	Precision (%)	F-Measure (%)
U-net	81.20	70.62	75.54
FPHBN	78.54	72.15	75.21
Our	87.66	77.48	82.26

**Table 4:** Comparison of the performance metrics of the first dataset crack samples for the four combinations.

Combination	Recall (%)	Precision (%)	F-Measure (%)
RGB	87.66	77.48	82.26
RGB + intensity	79.98	75.49	77.67
RGB + elevation	89.00	72.41	79.85
RGB + intensity + elevation	87.21	71.61	78.64

**Table 5:** Comparison of the performance metrics of the second dataset crack samples for the four combinations.

Combination	Recall (%)	Precision (%)	F-Measure (%)
RGB	88.54	86.61	87.56
RGB + intensity	86.93	75.65	80.90
RGB + elevation	90.09	77.21	83.15
RGB + intensity + elevation	88.77	76.47	82.16

**Table 6:** Comparison of the performance metrics of the second dataset sealed crack samples for the four combinations.

Combination	Recall (%)	Precision (%)	F-Measure (%)
RGB	80.07	90.17	84.82
RGB + intensity	83.22	92.41	87.57
RGB + elevation	83.99	92.59	88.08
RGB + intensity + elevation	84.22	92.44	88.14

**Table 7:** Comparison of the performance metrics of the third dataset crack samples for the four combinations.

Combination	Recall (%)	Precision (%)	F-Measure (%)
RGB	90.41	87.21	88.78
RGB + intensity	92.17	84.14	87.97
RGB + elevation	94.07	85.36	89.50
RGB + intensity + elevation	94.24	85.37	89.58

**Table 8:** Comparison of the performance metrics of the third dataset sealed crack samples for the four combinations.

Combination	Recall (%)	Precision (%)	F-Measure (%)
RGB	85.77	91.35	88.47
RGB + intensity	90.59	92.83	91.70
RGB + elevation	92.86	90.95	91.89
RGB + intensity + elevation	92.29	93.22	92.75

## Data Availability

The data presented in this study are not publicly available.
